# From COVID-19 Pandemic to Entrepreneurial Behavior: The Mediating Effect of Proactive Personality and the Moderating Role of Anticipated Regret

**DOI:** 10.3389/fpsyg.2022.838779

**Published:** 2022-06-06

**Authors:** Wang Jiatong, Majid Murad, Fu Bajun, Nausheen Syed, Muhammad Munir

**Affiliations:** ^1^College of Teacher and Education, Zhejiang Normal University, Jinhua, China; ^2^Department of Management Sciences, Muhammad Nawaz Sharif University of Engineering and Technology, Multan, Pakistan; ^3^School of Education, Shaoxing University, Shaoxing, China; ^4^Department of Business Administration, Government College Women University, Faisalabad, Pakistan; ^5^Department of Management and Administrative Sciences, University of Narowal, Norowal, Pakistan

**Keywords:** COVID-19 perception, proactive personality, entrepreneurial intention, entrepreneurial behavior, anticipated regret, university students

## Abstract

COVID-19 is a global public health issue that poses a challenge to the education sector. The pandemic has a devastating impact on student entrepreneurial behavior and their mental health. This study aimed to examine the impact of COVID-19 on the student entrepreneurial intention/behavioral model with a mediating effect of proactive personality and the moderating role of anticipated regret. The sample of the study comprised 345 university students from Pakistan. Data were collected using a self-report and other report survey questionnaires. The hypotheses were investigated using the partial least squares structural equation modeling (PLS–SEM) approach. According to the findings, COVID-19 perception has a negative and significant impact on the student entrepreneurial intention/behavior model. Meanwhile, findings show that a proactive personality significantly mediates the relationship between COVID-19 perception and entrepreneurial intention. The results show that anticipated regret moderates the relationship between entrepreneurial intention and entrepreneurial behavior in a favorable and significant way. Furthermore, discussion and implications were also discussed in this article.

## Introduction

Entrepreneurship is a determining factor for social and economic development, enhances the creation of wealth and value, and improves the well-being of nations (Bogatyreva et al., 2019; Li et al., 2020). Over the years, entrepreneurship has received a broad consensus in the academic and international communities on its importance; however, research on its relevance in uncertain and adverse situations is rather scarce (Jiatong et al., 2021a). COVID-19 is a dangerous disease caused by a new strain of coronavirus first identified in Wuhan, China, in December 2019 and after that, it is spread rapidly around the world (Deng and Peng, 2020). The WHO classified the coronavirus (COVID-19) outbreak as a pandemic on 11 March, 2020. Currently, there are more than 117 million cases being registered, and more than 2.59 million people died because of coronavirus (Khadka et al., 2020). COVID-19 pandemic has a strong effect on the psychical and psychological health of people. In Pakistan, more than 50% of people feel psychologically involved in this disease, decreasing their positive feelings and satisfaction with life (Ullah et al., 2020). This is echoed by the economic problems that can help influence people’s quality of life. Social and economic factors are the drivers of the conditions in which people live. Employment, safety, income, education, social support, and discrimination factors account for around 40% of all health (Jiatong et al., 2021b). The idea that social and economic factors are related to the health and satisfaction of the individual provides a starting point for an economy that is more focused on human well-being.

Previous research has shown that natural disasters, wars, terrorist acts, and pandemics damage countries’ growth and are connected with a drop in investment and gross domestic product (GDP) (Casady and Baxter, 2020; Filho et al., 2020). However, these challenges also affect the formation of new business development and other commercial activities (Lopes et al., 2021). The impact of COVID-19 epidemic has a significant impact on the potential entrepreneurs with entrepreneurial ambitions to create new enterprises (Cao et al., 2020; Hernández-Sánchez et al., 2020). Prior studies contend that entrepreneurial intent is the most significant element in new business growth (Barba-Sánchez and Atienza-Sahuquillo, 2018; Fragoso et al., 2019). According to Nowiński and Haddoud (2019), entrepreneurial intention is defined as the desire to establish a new business and pursue a profession other than that of a regular employee. In general, intentions are a good predictor of entrepreneurial activity (Neneh, 2019a,b). Similarly, Shirokova et al. (2016) imply that starting and running a new business does not always translate goals into actual behavior.

Furthermore, numerous researchers have discovered a clear relationship between entrepreneurial qualities and personality and entrepreneurial desire to start a new firm (Mei et al., 2017; Hu et al., 2018), but empirical studies on COVID-19 pandemics perception, environmental uncertainties, and entrepreneurship has received little attention in the literature. Hernández-Sánchez et al. (2020) explain how the proactive attitude influences COVID-19 perception and entrepreneurial intention. The literature has previously identified the link between proactive personality and entrepreneurial intention (Fuller et al., 2018; Liu et al., 2019; Mahmood et al., 2019). A proactive personality is associated with identifying the opportunity and finding the appropriate solution in uncertain environmental situations (Seibert et al., 1999; Yi-Feng Chen et al., 2021). Altinay et al. (2012) argue that if the ecological situations are uncertain and dangerous, it will affect the students’ entrepreneurial intention. Obschonka et al. (2018) found that individual and contextual factors appear to positively and substantially impact the intention/behavior model.

Previous studies argue that the relationship between intention and behavior is moderated by self-control, proactive personality, and sex (Van Gelderen et al., 2015; Shinnar et al., 2017; Neneh, 2019b). However, the anticipated regret is typically considered as a motivating element for entrepreneurial intention and action when outcomes are uncertain yet entrepreneurial behavior is socially essential. As a result, this study adds to the literature in the following ways: first, it extends the conceptual model (Neneh, 2019b) by implying anticipated regret as a moderator in the link between entrepreneurial intention and behavior. Second, this research contributes to the direct effect of COVID-19 pandemic perception on entrepreneurial intention toward entrepreneurial action in the study setting of Pakistani university students (Hernández-Sánchez et al., 2020). Third, this study contributes to the role of proactive personality as a mediating in the relationship with COVID-19 pandemic perception and entrepreneurial intention, adding to the body of knowledge in the fields of entrepreneurship and psychology (Li et al., 2020).

## Conceptual Model and Hypotheses Development

Theoretical models that analyze the entrepreneurial process emphasize the importance of personal, cognitive, and prescriptive factors to determine the probability that an individual would be willing to start a new business (Jiatong et al., 2021b). Among the main factors related to entrepreneurial intentions are personality traits associated with entrepreneurs (Fragoso et al., 2019). The results of previous studies suggest that personality traits substantially influence how entrepreneurs think, the objectives they set, and, through their actions, what they achieve (Cardon and Kirk, 2015; Henley et al., 2017). In particular, previous studies have established that an entrepreneur generally has an optimistic orientation toward the future and seems more capable of finding opportunities and achieving the desired objectives (Casson and Wadeson, 2007). This research will focus on four characteristics, namely, proactiveness, optimism, COVID-19 pandemic perception, and psychological need satisfaction, to quantify the relationship between these four traits of the students and their entrepreneurial intention. In summary, this approach will allow this research to determine the relative importance of the four business characteristics to predict the entrepreneurial intention of students in an adverse situation.

**FIGURE 1:**
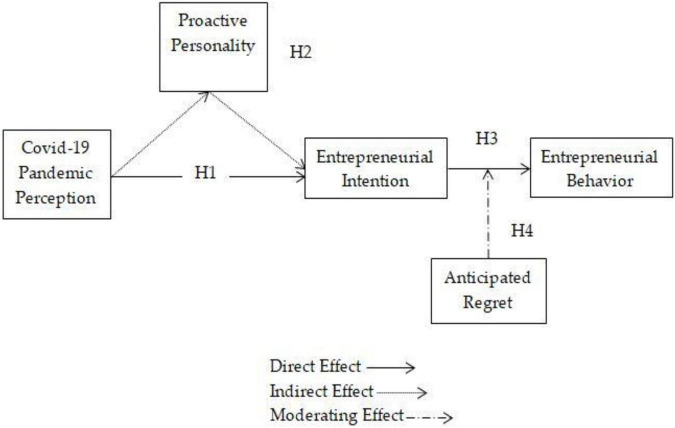
Conceptual model.

The research model is depicted in Figure 1. The model explains that COVID-19 pandemic perceptions negatively predict entrepreneurial intention, which positively indicates proactive personality on entrepreneurial intention, then anticipated regret moderates in the relationship between entrepreneurial intention and entrepreneurial behavior.

### COVID-19 Pandemic Perception and Entrepreneurial Intention

The prior study argues that COVID-19 pandemic perception has a negative and significant impact on entrepreneurial intention among business and economics students in Latin America (Hernández-Sánchez et al., 2020). Considering the role of social cognitive theory in entrepreneurship, the social and economic environment influences the attitude of the individuals through personal perceptions (Bacq et al., 2016). Moreover, perceptions are formed in the shape of unsafe and risky environments that affect individuals’ entrepreneurial intention to become entrepreneurs (Bergner et al., 2021). Ghosh (2018) suggests that terrorism attacks, neutral disasters, and uncertain environments harm countries’ growth and new business development activities. Bullough et al. (2014) argue that perceived danger, self-efficacy is negatively associated with entrepreneurial intention in Afghanistan. Therefore, based on the discussion, we posit that individuals with a higher COVID-19 pandemic perception are less likely to start a new business. Thus, we hypothesized that

**H1:** COVID-19 pandemic perception has a negative influence on the entrepreneurial intention.

### Mediating Effect of Proactive Personality

Proactive personality refers to identifying the opportunity, showing the advantage, and saving until important change happens (Buil et al., 2019). McCormick et al. (2019) define the importance of a proactive personality as “a person who can identify the opportunity in an uncertain environment.” Prior studies argue that individuals behave differently toward various environmental stimuli (Tolentino et al., 2014; Jiang, 2017). An individual with a high level of proactive personality will always carry about constructive enhancements. Hu et al. (2018) explored that more proactive people have more intention of business than other proper to start their own business. Literature advocates that COVID-19 perception and proactive personality significantly influence entrepreneurial intention in the context of Europe (Hernández-Sánchez et al., 2020). Several studies argue that proactive personality significantly influenced entrepreneurial intention and behavior even during natural disasters and economic crises (Neneh, 2019b; Li et al., 2020).

Kumar and Shukla (2019) found that their proactive personalities can predict the students’ entrepreneurial self-efficacy and entrepreneurial intention. Fuller et al. (2018) suggest that proactive people easily take entrepreneurial decisions to start a new business. Furthermore, Gupta and Bhawe (2016) explain that individuals who have more proactiveness and risk are more intend to have entrepreneurial intention to become entrepreneurs. Therefore, based on the discussion, we predict that more proactive individuals can manage the business development activities even in COVID-19 pandemic situation. Thus, we hypothesized that

**H2:** Proactive personality mediates in the relationship between COVID-19 pandemic perception and entrepreneurial intention.

### Entrepreneurial Intention and Entrepreneurial Behavior

In the domain of COVID-19 perception, the link between entrepreneurial intention and entrepreneurial conduct has received less attention (Van Gelderen et al., 2015; Shinnar et al., 2017). Entrepreneurial intention refers to an individual ability to start a new business, whereas entrepreneurial behavior refers to start an actual business (Barba-Sánchez and Atienza-Sahuquillo, 2018). Most of the previous studies discussed the theory of planned behavior as an extension of the theory of reasoned action for measuring the individual intention-behavior models (Mahmood et al., 2019; Neneh, 2019b). According to the theory of planned behavior intentions, an individual’s willingness to engage in entrepreneurial behavior or commitment to start a new business (Ajzen, 1991; Padilla-Angulo, 2017). Given the importance of this hypothesis, previous studies have found that intentions are the best indicator of entrepreneurial behavior (Shirokova et al., 2016; Neneh, 2019a). Moreover, Li et al. (2020) found that entrepreneurial purpose favorably influences entrepreneurial action, while proactive personality moderates this link. Because of this, people with higher levels of entrepreneurial inclination are more likely to engage in entrepreneurial behaviors and start their own businesses. Hence, we hypothesized that

**H3:** Entrepreneurial intention are positively associated with entrepreneurial behavior.

### Moderating Role of Anticipated Regret

Anticipated regret is associated with the negative feelings and emotions that an individual experiences before the expected result of their decision to perform a task (Neneh, 2019b). Prior research found that emotions played a vital role in stimulating innovative ideas and exploitation of that idea into reality. It helps individuals make entrepreneurial decision making to start a new business (Rodrigues et al., 2019). Metallo et al. (2021) explain that positive emotions create entrepreneurial passion among individuals while negative emotions create fear and grief to inhibit opportunity identification and exploitation among individuals to start a new business.

Therefore, anticipated regret pushes an individual to become an entrepreneur with passion and other business improvement activities (Pidduck et al., 2021). Moreover, studies found that individuals who had regrettable experiences from the past are more likely to involve in actual entrepreneurial behavior and avoid future regret that arises from a failure to act (Verkijika, 2019; Bouderbala, 2020). Thus, based on this discussion, people who have a lot of negative feelings are more likely to identify and take advantage of entrepreneurial possibilities, which could lead them to consider starting their own business (Ahmed et al., 2020). Hence, we hypothesized that

**H4:** Anticipated regret moderate in the relationship between entrepreneurial intention and entrepreneurial behavior.

## Materials and Methods

### Sample Design and Data Collection

Data were gathered from public and private sectors’ business and vocational university students of province, Punjab, Pakistan. Neneh (2019b) argued that there is an emerging and growing trend nowadays that business and vocational university students were engaged in forming new business startups. Scholars have stated that the most studies on students’ entrepreneurial goals have failed to investigate further the link between entrepreneurial intention and entrepreneurial action (Shirokova et al., 2016). Moreover, data were collected in two waves 5 months apart. A 5-month timeline was selected to reduce the possibility of different changes in intentions over a longer period.

In the first wave, we distributed 480 questionnaires among university students, and 400 received a participation rate of 83.3% (first wave). The objective of this study was to identify how entrepreneurial intentions translate into actual behavior. This study was interested in those students who did not actively participate in business gestation activities. A total of 35 students answered that they had already started a new business and hence were removed from taking part in the second wave of the study. After 5 months, the remaining 365 students were invited to participate in the second wave for data collection, and 345 responses were received in (second wave). Thus, the entrepreneurial intentions responses were measured in the first wave of data and entrepreneurial behavior responses in the second wave of data. The original draft of the questionnaire was in the English language because English is the official teaching language in the higher education sector of Pakistan. The profile of the respondents were (58% males and 42% females); age group (80% 18–30 years, 15.7% 31–40 years, and 4.3% were 41–above years); education (16.2% under-graduation, 42.6% graduation, 35.1% masters, and 6.1% Ph.D.).

### Common Method Bias

We used a variety of methods to avoid frequent method bias in the data. Harman’s single factor test was used first, and the principal component analysis revealed that no one component explained more than 50% of the overall variance in factor analysis. Moreover, we analyzed the correlation test among the variables, and the values were satisfactory and not exceeded the threshold value of 0.90 as suggested by Podsakoff and Organ (2016). Finally, we select self-report and other report surveys instead of self-reported data to avoid the issue of the halo effect and social desirability bias. Thus, this study has not any issue of common method and response biases.

### Measures

To measure students’ entrepreneurial intention, we adapted five items scale from the study Liñán and Chen (2009). Previous researchers widely used this scale (Jiatong et al., 2021a; Murad et al., 2021). A sample item is “I am ready to do anything to be an entrepreneur.” Moreover, to assess the proactive personality, we adapted six items’ scale from the study Mahmood et al. (2019). A sample item “I excel at identifying opportunities.” Furthermore, to assess the COVID-19 pandemic perception, we adapted six measurement constructs from the study Hernández-Sánchez et al. (2020). Sample items “The COVID-19 pandemic decrease my business opportunities.” Meanwhile, to measure the students’ entrepreneurial behavior, we adapted 10 items from the study Neneh (2019a). This scale was developed by the Global Entrepreneurship Monitor (GEM) and widely used by several researchers to evaluate the actual entrepreneurial behavior of the people (Neneh, 2019b; Li et al., 2020). A sample item “I have already purchased material for my business.” In addition, we adapted two measurement items from the study to assess the role of anticipated regret in the relationship between entrepreneurial intention/behavior relationships (Neneh, 2019b). A sample item “I feel upset if I did not engage in business start-up activities in the next 6 months.”

### Data Analysis Technique

We used Smart–PLS structural equation modeling technique to test the measurement and structural model of the study. This technique was widely used by prior researchers in social and management sciences (Hair et al., 2019). Smart-PLS–SEM method was used instead of the traditional covariance based/technique and that is due to the fact that CB–SEM requires a large sample size. The partial least squares (PLS) approach to SEM (PLS–SEM) is a suitable, favorable method or instrument used for estimating a complex, hierarchical model representing the credibility, and the methodology of soft modeling assumptions (Neneh, 2019b; Li et al., 2020).

## Results

### Measurement Model

The fitness of the measurement model was assessed through the Cronbach’s alpha, composite reliability, and convergent validity analysis. According to Henseler et al. (2014), the Cronbach’s alpha and composite reliability values should be >0.70, and values of composite reliability should be >0.80. Moreover, constructs convergent validity was assessed through average variance extracted. As suggested by Hair et al. (2012), the values of AVE should be >0.50. Hence, Table 1 shows that all the values of constructs reliability and validity were acceptable.

**TABLE 1:** Constructs factor loadings.

Constructs	Loadings	CA	CR	Rho_A	AVE	VIF
**Anticipated regret**		**0.892**	**0.949**	**0.895**	**0.903**	
	AR1	0.947				2.850
	AR 2	0.953				2.850
**COVID-19 pandemic perception**		**0.953**	**0.964**	**0.954**	**0.842**	
	COVID 1	0.947				4.356
	COVID 2	0.927				4.898
	COVID 3	0.920				4.338
	COVID 4	0.873				2.903
	COVID 5	0.919				4.407
**Proactive personality**		**0.869**	**0.903**	**0.906**	**0.619**	
	PP 1	0.749				2.647
	PP 2	0.730				2.537
	PP 3	0.873				3.415
	PP 4	0.852				3.155
	PP 5	0.902				4.003
	PP 6	0.911				4.548
**Entrepreneurial intention**		**0.926**	**0.945**	**0.935**	**0.774**	
	EI 1	0.865				2.889
	EI 2	0.787				2.175
	EI 3	0.885				3.217
	EI 4	0.935				4.864
	EI 5	0.919				4.319
**Entrepreneurial behavior**		**0.945**	**0.953**	**0.950**	**0.668**	
	EB 1	0.764				2.305
	EB 2	0.812				2.827
	EB 3	0.845				3.336
	EB 4	0.822				2.870
	EB 5	0.780				2.251
	EB 6	0.845				3.261
	EB 7	0.869				3.652
	EB 8	0.847				3.020
	EB 9	0.792				2.344
	EB 10	0.791				2.684

*CA, Cronbach’s alpha; CR, composite reliability; AVE, average variance extracted; VIF, variance inflation factor.*

Furthermore, discriminant validity was evaluated through Fornell and Larcker (1981) and the Heterotrait–Monotrait criterion. According to Fornell and Larcker (1981) square root of the AVE is discriminant validity, whereas the values of HTMT should be >0.85 (Henseler et al., 2014). Thus, results from Tables 2, 3 indicate that all the construct’s values met the discriminant validity criteria.

**TABLE 2:** Discriminant validity (Fornell–Larcker).

	AR	COVID	AR*EI and EB	EB	EI	PP
AR	0.950					
COVID	0.216	0.918				
AR*EI and EB	−0.226	−0.160	1.000			
EB	0.333	0.433	−0.025	0.817		
EI	0.325	0.406	−0.258	0.403	0.880	
PP	0.543	0.468	−0.021	0.481	0.456	0.787

*COV, COVID-19 pandemic perception; EI, entrepreneurial intention; PP, proactive personality; EB, entrepreneurial behavior; AR, anticipated regret.*

**TABLE 3:** HTMT ratio.

	AR	COVID	AR*EI and EB	EB	EI	PP
AR						
COVID	0.234					
AR*EI and EB	0.239	0.164				
EB	0.360	0.454	0.042			
EI	0.356	0.429	0.269	0.417		
PP	0.720	0.493	0.130	0.529	0.514	

*COV, COVID-19 pandemic perception; EI, entrepreneurial intention; PP, proactive personality; EB, entrepreneurial behavior; AR, anticipated regret.*

### Structural Model

**FIGURE 2:**
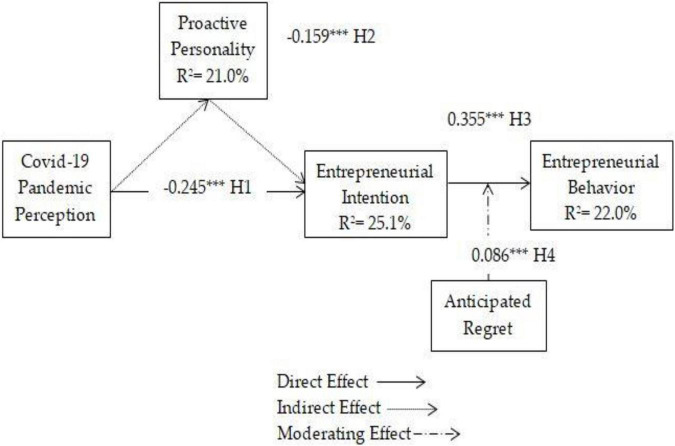
Structural model.

The fitness of the structural model was evaluated through the value of standardized root mean squares residual SRMR value. According to Henseler et al. (2014), a good model must have a >0.08 SMRM value. Our results show that the value of SMRM was 0.045, which indicates the adequate fitness of the model. Moreover, Figure 2 shows that the structural model explained *R*^2^ 22% variance in entrepreneurial behavior, *R*^2^ 25% explained variance in entrepreneurial intention, and *R*^2^ 21% explained variance in proactive personality. According to Chin (2010), the desired *R*^2^ should be greater than zero or one. Furthermore, to test the hypotheses, we have hypothesized four hypotheses, and the results of the hypotheses are shown in Table 4. The results of H1 indicate that COVID-19 pandemic perception has a negative and significant influence on entrepreneurial intention (β = −0.245^***^, *t* = 5.680, *p* = 0.001). Therefore, H1 was accepted. Findings of H2 show that proactive personality has a positive and significant indirect effect on the relationship between COVID-19 pandemic perception and entrepreneurial intention (β = −0.159^***^, *t* = 5.486, *p* = 0.001). So, H2 was supported. Meanwhile, the results of H3 indicate that entrepreneurial intention positively associated with entrepreneurial behavior (β = 0.355^***^, *t* = 6.843, *p* = 0.001). Consequently, H4 was accepted. In addition, to test H4, findings illustrate that anticipated regret positively moderates the relationship between entrepreneurial intention and entrepreneurial behavior (β = 0.086^***^, *t* = 2.853, *p* = 0.005). Hence, H4 was also supported.

**TABLE 4:** Hypotheses testing.

Hypotheses	Relationships	β	T	*p*	Decision
H1 Direct effect	COV → EI	−0.245***	5.680	0.001	Accepted
H2 Indirect effect	COV → PP → EI	−0.159***	5.486	0.001	Accepted
H3 Direct effect	EI → EB	0.355***	6.843	0.001	Accepted
H4 Moderating effect	AR*EI and EB	0.086***	2.853	0.005	Accepted

*COV, COVID-19 pandemic perception; EI, entrepreneurial intention; PP, proactive personality; EB, entrepreneurial behavior; AR, anticipated regret.*

**p < 0.05, ***p < 0.001.

## Discussion

The purpose of this study was to contribute to COVID-19 pandemic perception on entrepreneurial intention and entrepreneurial behavior among university students in Pakistan who wish to pursue a career in entrepreneurship. Regarding H1, we hypothesized that the COVID-19 pandemic was negatively associated with entrepreneurial intention. Our findings support this rationale that the perception of COVID-19 pandemic played a negative role in the development of the entrepreneurial process of university students. Our results are consistent with the prior study Hernández-Sánchez et al. (2020) who found similar findings in the context of Latin American business and economics students on COVID-19 perception and entrepreneurial intention. This study model, and the results obtained, is a first attempt to close the gap in our knowledge of what drives entrepreneurship in highly adverse conditions (for example, in economies subject to pandemics).

Concerning H2, according to the results of this study, proactive personality would operate as a mediator between COVID-19 pandemic perception and entrepreneurial intention. Our study adds to the previous literature on personality and entrepreneurship’s mediation outcome of proactive personality, although some past studies reported a direct influence of proactive personality on entrepreneurial intention (Hu et al., 2018; Kumar and Shukla, 2019). As such, according to this study, influencing the entrepreneurial intention of students (potential entrepreneurs), who are hampered by the pandemic situation, means analyzing the psychological and social factors that influence intentions and understanding how they relate to entrepreneurial behavior in the practice. Therefore, it is highly important to appreciate proactive traits in students so that they can think of entrepreneurship as a possible career option. Furthermore, our research adds to the body of knowledge by concluding that people with a high level of proactive personality are more likely to spot changes in uncertain situations.

Regarding H3, we found that entrepreneurial intention would lead to actual entrepreneurial behavior. This result is in line with previous researchers, who found similar entrepreneurial intention/behavior models results and suggest that students with entrepreneurial intention usually start their new businesses (Shirokova et al., 2016; Fuller et al., 2018; Neneh, 2019a). Concerning H4, our results show that anticipated regret positively moderates the relationship between entrepreneurial intention and entrepreneurial behavior. This finding is similar to Neneh (2019b) and Ahmed et al. (2020), who indicates that those with a high amount of anticipated regret are most likely to engage in entrepreneurial activities. This study presents an innovative approach because it examines the psychological aspects of entrepreneurial intention in a pandemic situation. Furthermore, our work confirms previous studies that also show the positive role of need satisfaction, which acts as an accelerator of an entrepreneurial initiative by increasing entrepreneurial intention. In this way, it joins the growing literature that explores how basic psychological needs act as motivational drives that positively influence one’s future, including career choice.

## Implications

Our study suggests important implications for the higher education sector of Pakistan into the following perspectives, who want to promote entrepreneurship in particularly difficult situations such as pandemic and economic crises. To respond efficiently to the crises, first it is essential to consider its effects on entrepreneurship. This will help students act rapidly and emerge stronger from COVID-19 pandemic perception of the entrepreneurial startup process. First, regarding the direct influence of COVID-19 pandemic on entrepreneurship, this study provides a moment of reflection about the proactive personality could affect individuals’ lives and their happiness because when the environmental situation is uncertain, in a pandemic crisis, persons with a high level of proactive personality are more likely to call on their innovative abilities and establish entrepreneurial intentions. A proactive personality emphasizes strategic goals and prolific activities, even when times are tough. Second, entrepreneurship study courses would be offered within schools and universities to promote the entrepreneurial culture at the national and international levels. Educators should provide assistance and conduct training programs for students and encourage curriculum projects that raise useful personal abilities to make entrepreneurial mindset at the university level. Third, according to this research, educators should provide students with online training and webinars that foster entrepreneurial purpose and urge them to start a new business while also considering the potential for future regret. Finally, entrepreneurial coaches should prosper to encourage aspiring entrepreneurs to think about the probability of regretting their inaction if it comes to pass.

## Conclusion

This study is important in the current instant of international economic crisis as a consequence of COVID-19 pandemic as it involves the production structures of many countries, which are at risk of an implosion in terms of economic growth, the most evident effects of which they can already be seen in the processes of a reduction in the labor market of numerous employees, and by the increasing phenomena of discouragement and/or resignation from job participation, especially in younger generations.

Our study found that the perception that university students have of the COVID-19 pandemic is decreasing their intentions to start a business, with repercussions on their psychological needs. How can we solve this? In our study, we have considered two personality traits that manifest themselves as keys to enhancing the intention to undertake in this specific situation, e.g., proactiveness. This trait can dampen the effect of COVID-19’s negative perception on the intention to start a business, and also enhance the well-being and mental health of the young people to be able to start a new business.

## Limitations and Future Directions

Future researchers can learn from our findings of the study’s limitations and potential directions for future research. We used an exploratory research design with a self-administered questionnaire and two waves of data collection from the respondents. According to Hernández-Sánchez et al. (2020), the use of self-report data and one-or-two times collected data raises the question about the variation of the method. Therefore, longitudinal research might be conducted on these constructs to generalize the results in different study settings. Second, we gathered data from the public and private sectors’ university students of Punjab, Pakistan, with minor sample size. We recommend that future researchers conduct an empirical study related to the impact of COVID-19 pandemic on social entrepreneurship with the mediating effect of resilience using different country samples. Future research could also take COVID-19 pandemic perception and cultural lifestyle on entrepreneurship.

## Data Availability Statement

The raw data supporting the conclusions of this article will be made available by the authors, without undue reservation.

## Ethics Statement

The studies involving human participants were reviewed and approved by the Ethics Committee of the Muhammad Nawaz Sharif University of Engineering and Technology, Multan, Pakistan. Written informed consent to participate in this study was provided by the participants.

## Author Contributions

WJ and MaM proposed the research, analyzed the experimental results, and wrote the manuscript. FB, NS, and MaM designed and carried out the experiments and extensively edited the manuscript. All authors contributed to the article and approved the submitted version.

## Conflict of Interest

The authors declare that the research was conducted in the absence of any commercial or financial relationships that could be construed as a potential conflict of interest.

## Publisher’s Note

All claims expressed in this article are solely those of the authors and do not necessarily represent those of their affiliated organizations, or those of the publisher, the editors and the reviewers. Any product that may be evaluated in this article, or claim that may be made by its manufacturer, is not guaranteed or endorsed by the publisher.
